# How do visual and smartphone camera-based shoulder ranges of motion compare?

**DOI:** 10.1016/j.jseint.2025.05.026

**Published:** 2025-06-10

**Authors:** Wolbert van den Hoorn, Maxence Lavaill, Freek Hollman, Roberto Pareyón Valero, François Bruyer-Montéléone, Kenneth Cutbush, Ashish Gupta, Graham Kerr

**Affiliations:** a School of Exercise & Nutrition Sciences, Queensland University of Technology, Brisbane, QLD, Australia; b Queensland Unit for Advanced Shoulder Research, Brisbane, QLD, Australia; c School of Mechanical, Medical and Process Engineering, Queensland University of Technology, Brisbane, QLD, Australia; d Centre for Biomedical Technologies, Queensland University of Technology, Brisbane, QLD, Australia; e School of Medicine, The University of Queensland, Brisbane, QLD, Australia; f Greenslopes Private Hospital, Brisbane, QLD, Australia

**Keywords:** Thoracohumeral, Range of motion assessment, Validity, Pose estimation, Shoulder, Smartphone

## Abstract

**Background:**

Objective assessment of functional shoulder range of motion (ROM) is crucial for evaluating shoulder interventions and guiding rehabilitation. The goniometer is the clinical standard, but due to practicality, visual estimation is often used despite its lower reliability. Recently, smartphone video-based assessment using two-dimensional (2D) pose estimation models has emerged as a potential objective alternative. This study aimed to compare 2D-pose–based ROM assessment with visual estimation and examine inter-observer agreement.

**Methods:**

Seventeen individuals (8 females, 9 males) with normal, pain-free shoulder function were assessed for active ROM in abduction, flexion, extension, external rotation (ER) in two positions (ERI & ERII), and functional internal rotation (FIR). 2D videos from three smartphones were used to estimate shoulder ROM, while two othopedic surgeons visually estimated ROM. For each movement, participants performed six repetitions, three at maximum and three less than maximum ROM (self-selected). Mixed effects models assessed the relationship between 2D-pose–based and visual-based ROM, with visual observer as fixed factor and visual estimates × observer interaction. The coefficient of determination (R^2^) from these mixed effects models assessed consistency, and smallest detectable difference was used to determine agreement.

**Results:**

Consistency between 2D-pose and visual estimates was excellent for abduction (R^2^ = 0.99), flexion (R^2^ = 0.95), and ERII (R^2^ = 0.86), good for extension (R^2^ = 0.69) and ERI (R^2^ = 0.73), and fair for FIR (R^2^ = 0.52). Smallest detectable difference values ranged from 4.4° to 7.9°. Agreement varied by movement type and observer, with significant visual estimates × observer (*P* < .003) interaction effects for abduction and flexion: both observers reported higher ROM values than 2D-pose near end-ROM (∼3-4°) with observer 2 reporting lower values than observer 1 (∼15°) at smaller ROM (<60°). 2D-pose estimates were higher (∼20°) for extension at low ROM (<45°) than visual estimates. 2D-pose estimates were lower (∼30°) for ERI at high ROM (>45°) than visual estimates. Visual observers agreed on extension, ERI, ERII, and FIR estimates but disagreed on abduction and flexion estimates.

**Conclusion:**

2D-pose–based estimates of shoulder ROM were consistent with visual estimates for most movements, though discrepancies existed at specific ROM levels and between observers. The higher resolution estimates of 2D-pose suggests it could reduce observer variation, making it a promising alternative for clinical and research settings. However, further refinement is needed for movements like ERI and FIR using both methods. These findings highlight the importance of method consistency in assessing shoulder ROM and the potential benefits of automated methods for more consistent evaluations.

Objective assessment of functional shoulder range of motion (ROM) is critical for evaluating shoulder interventions and guiding rehabilitation to optimize patient outcomes.^6^^,^^16^ The goniometer method is considered the clinic standard.^18^ However, due to time constraints and ease of use, active ROM is often estimated via visual observation.^14^^,^^22^ The reliability of both methods is suboptimal for assessing intervention efficacy^19^^,^^20^^,^^22^ because of variations in assessed ROM between and within observers,^10^^,^^25^ and/or the ability to detect small differences/changes via visual observation.^24^ Smartphone video-based assessment of ROM using machine learning models^1^ to automatically identify body landmarks—(two-dimensional [2D] pose)—presents a potential objective method for estimating active ROM.^11^ While previous studies have compared 2D pose against gold-standard tools such as three-dimensional (3D) motion capture,^11^ real-world adoption in clinical settings will also depend on how these tools align with current practice. In many clinics, particularly orthopedic settings, visual estimation remains a commonly used method. Therefore, evaluating the relationship between 2D-pose–based ROM estimates and clinician visual assessment is clinically relevant. Yet, it remains unclear how visual and 2D-pose video-based methods compare.

The resolution and accuracy of methods estimating ROM should be at least smaller than the minimal clinically important difference (MCID).^21^ The MCID represents the threshold at which a change in an outcome measure is meaningful to patients.^12^ For active shoulder ROM, MCID ranges from 3° to 12°, depending on the type of shoulder movement.^21^ These thresholds indicate the need for high-fidelity methods. Visual estimates of shoulder ROM are often given in 10° increments, suggesting a maximum resolution of 10°. In contrast, 2D-pose–based methods could provide higher resolution estimates of shoulder ROM.

It is critical that alternative methods to visual estimation of shoulder ROM measure the same construct. Studies suggest a close relationship between visual estimation and universal goniometer^14^ or other pose-based methods,^8^^,^^24^ albeit with some variation in methods and accuracy. The Microsoft Kinect camera system (now named Azure Kinect; Microsoft Corp., Redmond, WA, USA) has improved ROM accuracy relative to visual observation^8^^,^^24^ by using automated identification of key body landmarks. However, it requires a specialized camera setup (multiple Kinect cameras) connected to a personal computer, making it less ideal for out-of-clinic use. Smartphone-based methods, which are widely available and easy to use, offer an alternative. They process data on the phone but are limited to a 2D plane view. Using a more objective 2D-pose method may also help reduce variations both between and within observers. It is important to compare visual assessments with 2D-pose–based methods to understand their relative accuracy.

The first aim of our study was to determine the relationship between 2D-pose and visual estimation of shoulder ROM for standard clinical active shoulder ROM tests. The second aim was to determine the agreement between two expert observers. Data were collected from individuals with normal, pain-free shoulder function. We hypothesized that visual and 2D-pose–based estimations of active shoulder ROM would agree and be consistent between methods, and that the two observers would agree.

## Materials and methods

### Participants

Seventeen individuals with normal pain-free shoulder function volunteered. The group consisted of 8 females and 9 males with a mean (standard deviation) age of 31 (7) years, height of 1.72 (0.10) m, and weight of 68 (13) kg. Data from this cohort has been published.^11^ Participants were recruited from the local community via convenience sampling. Participants were excluded if they had any history of shoulder dislocations/perceptions of subluxations, shoulder pain of any origin, or limited shoulder function that caused them to seek care within the previous 6 months; if they were less than 18 years of age; unable to understand written or spoken English; or unable to provide written informed consent. Ethics approval was obtained from the local University Human Ethics Committee (QUT ethics number #2000000470).

### Instruments

**Figure 1:**
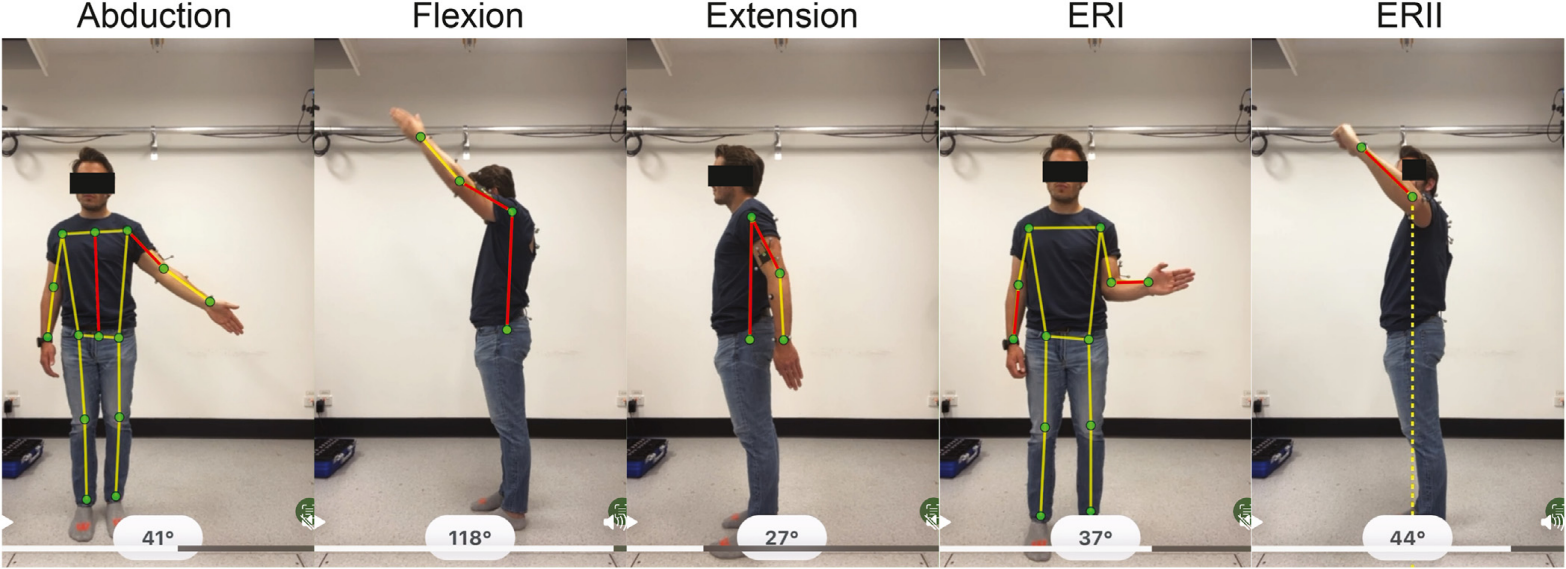
Shoulder movements. From left to right: abduction, flexion, extension, ERI, and ERII. Participant positioning relative to the camera is shown: facing the camera for abduction and ERI; side view for flexion, extension, and ERII. In ERI, participants kept the elbow at 90° flexion and maintained elbow–torso contact while externally rotating; In ERII, participants held 90° shoulder abduction and elbow flexion. ERI angle was computed using inverse sine based on ratio of horizontal wrist–elbow projection over contralateral wrist–elbow length. ERII angle was calculated using inverse tangent relative to the global horizontal. All other shoulder angles were calculated using inverse tangent between relevant segments (*shown in red*). Note that the public version of the app does not display these landmarks or angle values. *ERI*, external rotation in position I; *ERII*, external rotation in position II.

**Figure 2:**
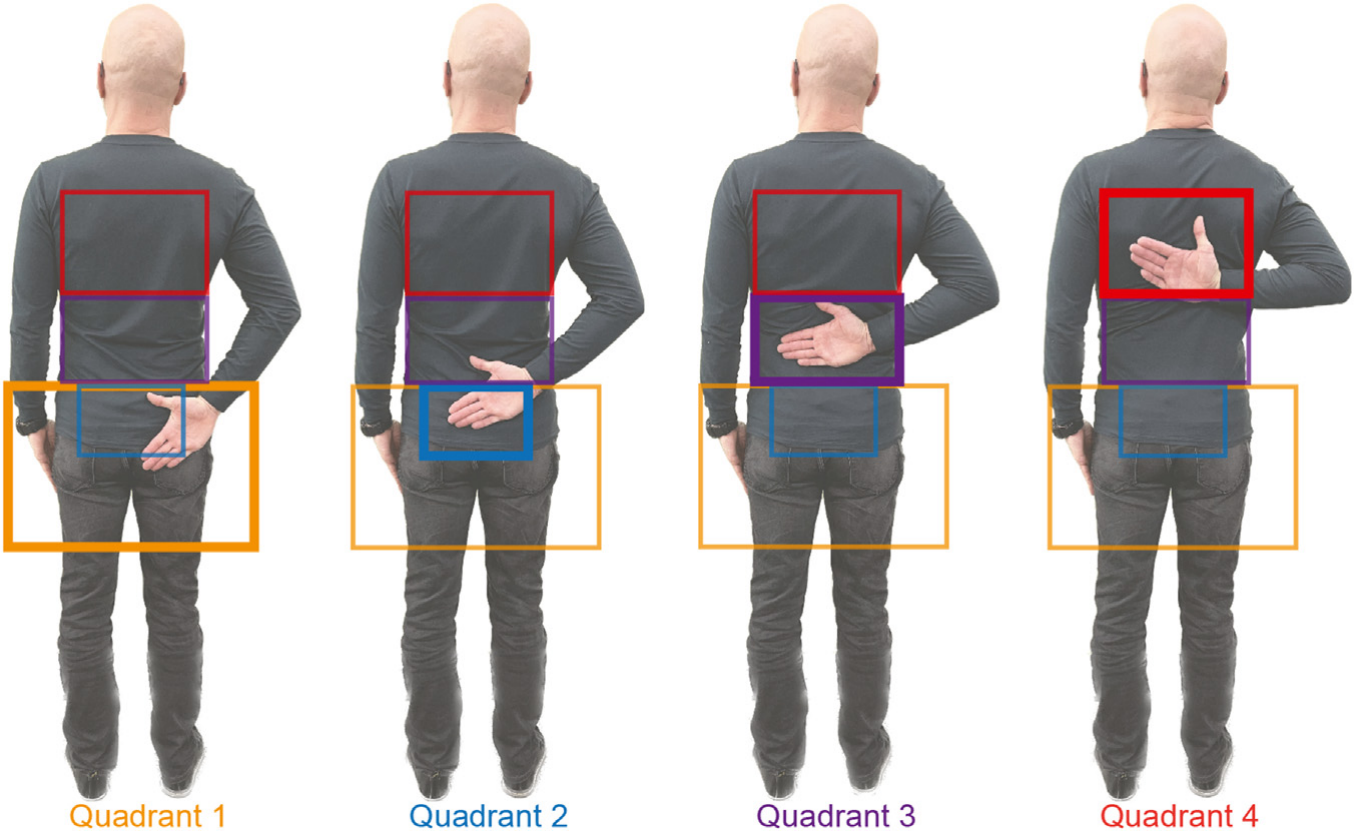
Functional internal rotation. Definition of zones for functional internal rotation measured using 2D-pose–based wrist position are shown. Quadrants increase gradually with higher functional internal rotation range as measured by the Range of Motion Models in the mymobility App. *2D*, two dimensional.

**Figure 3:**
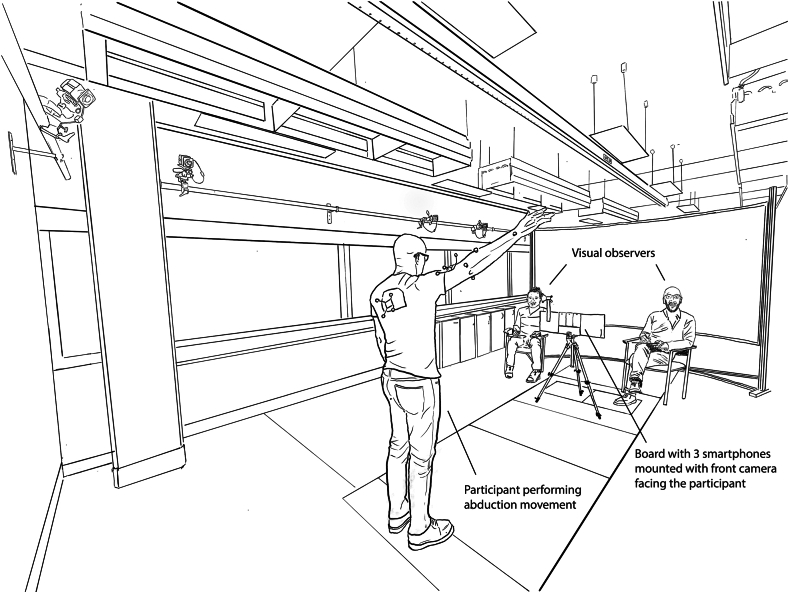
Measurement setup. Upper limb movements were concurrently recorded using 3 iPhones (mounted on a board) and viewed by two surgeons (visual observers).

Active shoulder ROM was assessed concurrently using 2D-pose and visual estimation during abduction, flexion, extension, external rotation in position I (ERI) & II (ERII) (Fig. 1), and functional internal rotation (FIR) (Fig. 2). The front-facing camera of 3 iPhones (2 × iPhone 13, and 1 iPhone 13 Pro [Apple, Inc., Cupertino, CA, USA],) was used to sample the 2D-pose at 30 samples/s using the mymobility care management platform Skeletal Tracking Shoulder Range-of-Motion Assessments feature (Zimmer Biomet, Warsaw, IN, USA). The iPhone 13 and iPhone 13Pro front-facing camera hardware are identical—both contain a 12-MP resolution with f/2.2 aperture camera. Using the Apple Vision framework (Apple, Inc., Cupertino, CA, USA),^1^ body landmarks are detected from the 2D videos, from which shoulder ROM was estimated (v3.7.0; Zimmer Biomet, Warsaw, IN, USA). The three phones were mounted on a board, aligned with gravity using the built-in Measure App, and positioned to ensure the front-facing camera could view the participant (Fig. 3). The distance between the phones and the participant (1.86 [0.14] m) was optimized so that the whole body remained within camera view during shoulder movements. We ensured the phone orientation was perpendicular to the movement plane by manually aligning the phone to the room.

The 2D-pose data were processed locally on the phone to determine thoracohumeral angle (see Fig. 1 for key body landmarks used to determine ROM). The Range of Motion Model of the mymobility App usually provides the maximum achieved ROM during a trial only. For this study, the time-series data for each shoulder movement type were uploaded to a dedicated server for further off-line processing. We observed some fluctuations in the 2D-pose elbow and wrist positions during ER-I; therefore, a 10th-order median filter was applied to the angular data (positional data were not exported) prior to further processing. The ROM of all shoulder movement types were expressed in degrees, except for FIR, which the Range of Motion Model measured in 4 zones (see Fig. 2 for definitions).

Two shoulder surgeons served as visual observers and were seated on the left and right side of the phones, approximately 2.5 m from the participant (Fig. 3). They visually estimated the shoulder ROM for each movement and recorded their estimates. We ensured that observations were blinded, ie, there was no oral or visual communication between the observers and the phones throughout the entire duration of the study. After completing the observations, visual observation data were entered into an electronic spreadsheet for further analysis.

### Procedures

#### Visual observation of shoulder range of motion

Participants were instructed to perform 3 repetitions of their full ROM (each starting from neutral rest position) for each shoulder movement type (order randomized). Directly afterwards, participants performed 3 additional repetitions with self-selected ROM, also starting from rest position, that were intentionally less than their maximum available ROM to enable comparison across different ROM levels. At each repetition, the participant briefly held (∼2 s) the reached ROM position to allow the angle to be estimated and recorded by the observers. For the FIR, the visual observers noted the vertebral level reached by the participant's thumb. These values were converted to an overall vertebral number for analysis, with T8 corresponding to the 15th spinal vertebra, for example.

The 2D-pose–based shoulder angle measured by the 3 phones was determined at the achieved ROM at each repetition (Fig. 3) for each shoulder movement type. The data were organized along with the corresponding visual estimates from the two observers, for further statistical analysis.

FIR was expressed in different units for 2D-pose (in zones) and visual assessment (in vertebral level). To enable visual assessment comparison with 2D-pose, visual assessment was scaled as follows:

$$visual_{scaled}=visual-\min\left(vertebra\right)*\frac{\left(\max\left(zone\right)-\min\left(zone\right)\right)}{\left(\max\left(vertebra\right)-\min\left(vertebra\right)\right)}+\min\left(zone\right)$$

where max and min reached zone across the participants were 4 and 2, respectively, and max and min vertebra number were 27 and 11, respectively.

### Statistical analysis

Statistical analysis was performed using Stata (version 17; StataCorp, College Station, TX, USA), with significance level set at *P* < .05.

### Aim 1: the relationship between two-dimentional pose and visual estimates of shoulder range of motion

#### Convergent validity

The relation between 2D-pose estimates (independent variable) and visual observation (dependent variable) was assessed using linear mixed models for each movement type separately. Visual estimates were entered as covariate, with observer and visual estimates × observer interaction included as fixed effects. Participants were entered as random intercepts. Point estimates and their 95% confidence intervals (CIs) were determined using the maximum likelihood function. In accordance with the international COnsensus-based Standards for the selection of health Measurement INstruments (COSMIN guidelines^15^), the slope of the regression reflects convergent validity; eg, how changes in the reference measure (visual estimates) correspond to changes in the test measure (2D pose).

Because 2D-pose–based FIR is a categorical variable (zones 1-4), 2D-pose data were modeled with generalized estimating equations using a Poisson's distribution, identity link, exchangeable within-participant correlation structure, and robust estimation of CIs. Like the linear mixed models, scaled visual data were entered as covariate; observer was entered as a fixed factor, and visual estimates × observer interaction was included.

### Consistency between visual and two-dimensional pose–based range of motion

The coefficient of determination (R^2^) of the models was determined. R^2^ indicates the consistency between two measures. An R^2^ of 1 signifies that all the variance in an outcome measure is directly associated with the variance in the other measure. This is independent of the magnitude of the variation and does not indicate agreement. R^2^ values were interpreted as follows: values of <0.4, between 0.4 and <0.6, between 0.6 and <0.75, and >0.75 were classified as poor, fair, good, and excellent, respectively.^4^

**Figure 4:**
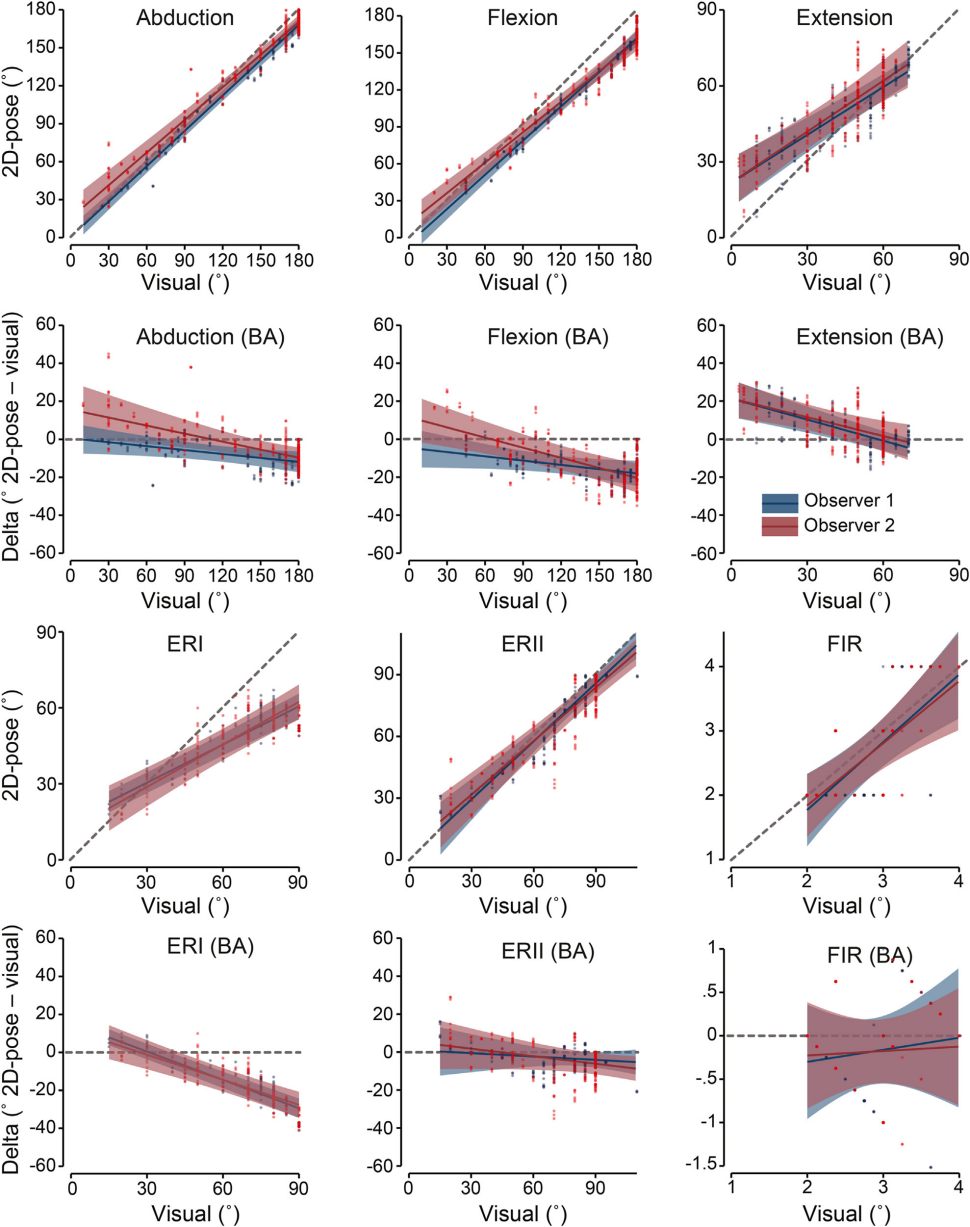
Comparisons between 2D-pose and visual estimation of active shoulder ROM. The linear relationship and the 95% CI of the model (see statistical analysis for more detail) between 2D-pose and visual estimation of each observer is provided (*blue* = observer #1, *red* = observer #2), and individual data points (scatter) for abduction, flexion, extension, ERI ERII, and FIR. The diagonal dashed lines indicate perfect agreement (y = x). The BA plots show the difference (bias) (Delta = 2D-pose-visual) vs. visual, and the 95% CI of the model is displayed below each linear relationship between 2D-pose and visual plot and represents the LoA based on the 95% CI from the linear mixed model. The horizontal dashed line indicates perfect agreement (Delta = 0). *ROM*, range of motion; *CI*, confidence interval; *ERI*, external rotation in position I; *ERII*, external rotation in position II; *FIR*, functional internal rotation; *BA*, Bland–Altman; *LoA*, limits of agreement.

The smallest detectable difference (SDD), an additional measure of consistency, was determined as the average of the 95% CI level of the predicted error values from the observed 2D-pose shoulder movement range (SDD_95_). Minimum and maximum bounds of the 95% CI were also extracted to characterize variation across the ROM spectrum. While this estimate reflects the precision of the modeled difference between visual and 2D-pose methods, it does not constitute the full limits of agreement (LoA), which require adding the estimated bias at each ROM level. Traditional LoA assume constant bias across the measurement range; however, in our case, bias systematically varied with ROM, as shown in the delta models (see Bland–Altman plots in Fig. 4). Applying LoA under nonconstant bias would overestimate disagreement and obscure the ROM-dependent nature of measurement error. Therefore, the SDD_95_ provides a more appropriate measure of error consistency, because a consistent difference (ie, independent of bias) would be required to establish a narrow CI of the difference. SDD_95_ is interpreted as the value above which a change in 2D-pose–based ROM estimation is beyond potential measurement error.^23^

### Agreement (model-based Bland–Altman analysis)

In line with Bland–Altman principles, agreement between 2D-pose and visual observations was assessed by modelling the difference (bias) between the two methods. The difference was calculated as: δ=2Dpose−visual. This model used the same structure as described above, with participants as random intercepts. Rather than calculating a single average bias, this model-based approach allowed us to examine whether bias varied systematically across the ROM, providing a more detailed and accurate characterization of agreement between methods.

### Aim 2: the agreement between observers

Differences between observers were determined from the above-described statistical models. In the case of significant main effect of observer, the overall marginal effect between observers with 95% CI was extracted. In the case of a significant visual estimates × observer interaction, the marginal effects between observers with 95% CI were extracted across the assessed ROM values.

## Results

Not all participants were assessed by both observers due to circumstances. N = 13 were assessed by both observers concurrently, and n = 3 participants were assessed by a single observer (observer 1: n = 1; observer 2: n = 2). N = 1 was not visually assessed and was therefore excluded from further analysis. FIR was only recorded for the last 11 participants.

The total number of simultaneous ROM estimates made by the observers was 432. Observer 1 made 468, and observer 2 made 486 ROM estimates.

### Aim 1: the relationship between 2D-pose and visual estimates of shoulder ROM

#### Consistency

The consistency between 2D-pose and the observers was excellent for abduction (R^2^ = 0.99), flexion (R^2^ = 0.95), and ERII (R^2^ = 0.86); good for extension (R^2^ = 0.69) and ERI (R^2^ = 0.73); and was fair for FIR (R^2^ = 0.52). See Table I for more details. The mean SDD_95_ values across the shoulder movement types ranged between 4.4° and 7.9° (see Table II for more details) with, in general, lower values of SDD_95_ for observer 1 vs observer 2. The SDD_95_ for FIR was approximately 0.5 zones for both observers (Table II).

**Table I:** Model coefficients describing the relationships between 2D-pose and observers for all shoulder movements.

Movement	Model	Intercept				Slope (visual covariate)				Interaction		
		Observer 1	*P* value	Observer 2	*P* value	Observer 1	*P* value	Observer 2	*P* value	Observer difference (O2−O1)	*P* value	R^2^
Abduction	Main model	0.55 (−3.50, 4.61)	.789	15.63 (8.17, 23.08)	<.001	0.93 (0.90, 0.96)	<.001	0.86 (0.81, 0.91)	<.001	−0.07 (−0.12, −0.02)	.003	0.99
	Delta model					−0.07 (−0.10, −0.04)	<.001	−0.14 (−0.19, −0.09)	<.001			0.48
Flexion	Main model	−4.50 (−9.85, 0.85)	.099	11.53 (5.25, 17.82)	<.001	0.92 (0.88, 0.97)	<.001	0.82 (0.78, 0.86)	<.001	−0.10 (−0.15, −0.06)	<.001	0.95
	Delta model					−0.08 (−0.11, −0.03)	<.001	−0.18 (−0.22, −0.14)	<.001			0.74
Extension	Main model	21.36 (13.33, 26.40)	<.001	21.42 (16.35, 26.50)	<.001	0.63 (0.57, 0.69)	<.001	0.67 (0.57, 0.77)	<.001	0.04 (−0.07, 0.15)	.463	0.69
	Delta model					−0.37 (−0.43, −0.31)	<.001	−0.33 (−0.43, −0.23)	<.001			0.38
ERI	Main model	15.34 (13.04, 17.64)	<.001	11.97 (6.60, 17.35)	<.001	0.50 (0.48, 0.53)	<.001	0.56 (0.49, 0.63)	<.001	0.06 (−0.004, 0.12)	.070	0.73
	Delta model					−0.50 (−0.52, −0.47)	<.001	−0.44 (−0.51, −0.37)	<.001			0.75
ERII	Main model	1.21 (−6.73, 9.14)	.766	5.74 (−1.93, 13.40)	.142	0.94 (0.85, 1.04)	<.001	0.87 (0.79, 0.95)	<.001	−0.07 (−0.20, 0.05)	.251	0.86
	Delta model					−0.06 (−0.15, 0.04)	.227	−0.13 (−0.21, −0.05)	.001			0.12
FIR	Main model	−0.34 (−1.11, 0.42)	.379	−0.10 (−0.82, 0.62)	.788	1.06 (0.80, 1.31)	<.001	0.97 (0.71, 1.23)	<.001	−0.09 (−0.37, 0.19)	.540	0.52
	Delta model					0.14 (−0.18, 0.46)	.398	0.05 (−0.22, 0.33)	.716			0.01

*ERI*, external rotation in position I; *ERII*, external rotation in position II; *FIR*, functional internal rotation; *ROM*, range of motion; *CI*, confidence interval; *2D*, two dimensional.

In line with Bland–Altman principles, the delta (visual: 2D-pose) model represents the bias between the visual and 2D-posed–based range of motion estimates across the ROM. This model allows bias to be evaluated as a function of shoulder angle, accounting for ROM-dependent differences.

Intercepts and interaction coefficients (95% CI) are the same between the main model and delta models and are reported once for clarity. *Intercept interpretation:* Represents the bias (visual: 2D-pose) at 0°, where a positive value indicates that 2D-pose estimates greater ROM, than visual assessment. *Slope interpretation:* Describes how each 1° increase in visual estimate corresponds to a change in 2D-pose estimate. *Interaction interpretation:* Indicates whether the slope differs between visual observers. A negative interaction term reflects a lower slope for observer 2 compared to observer 1; this was statistically significant for abduction and flexion. R^2^ reflects the consistency between two measures (see statistical analysis section for more information).

**Table II:** SDD_95_ 95% CI.

	SDD_95_ range		
	mean	min	max
Abduction			
Obs 1	5.1	4.1	7.4
Obs 2	7.8	4.8	13.6
Flexion			
Obs 1	6.2	4.7	9.7
Obs 2	7.9	6.4	11.5
Extension			
Obs 1	6.7	5.1	9.4
Obs 2	7.6	6.6	9.4
ERI			
Obs 1	4.4	4.1	5.1
Obs 2	6.9	6.2	8.9
ERII			
Obs 1	6.6	3.7	12.7
Obs 2	7.8	5.6	12.7
FIR			
Obs 1	0.52	0.38	0.80
Obs 2	0.47	0.37	0.67

*ERI*, external rotation in position I; *ERII*, external rotation in position II; *FIR*, functional internal rotation; *Obs*, visual observer; *ROM*, range of motion; *CI*, confidence interval; *SDD*, smallest detectable difference.

In line with Bland–Altman principles, the SDD_95_ represents the smallest detectable difference and is conceptually related to the limits of agreement. Values are derived from the mean 95% CI of the residual error from the Delta scores' linear mixed model across the ROM (see Table I and Bland–Altman plots in Fig. 4). Minimum and maximum values across the ROM, range are also reported.

#### Agreement (bias)

For abduction and flexion, the agreement between 2D-pose and visual observations was dependent on the observer. Both a main effect of observer (*P* < .01, Table I) and visual estimates × observer interaction (*P* < .01) were observed. Compared to 2D-pose, while both observers tended to report higher ROM values than 2D-pose approaching end-ROM (>150°, *P* < .001), observer 2 reported lower values than observer 1 at smaller ROM (<50°, see agreement between observers for more details).

For the extension, ERI, ERII, and FIR movements, neither main effect of observer (*P* > .17, Table III), nor visual estimates × observer interaction (*P* > .07, Table I) were observed. Therefore, agreement between 2D-pose is compared against the estimates of both observers. For both extension and flexion movements, the agreement between 2D-pose and observers depended on ROM. For extension values below ∼45°, 2D-pose estimates were greater than those made by the observers (Fig. 4). For ERI values greater than ∼40°, 2D-pose estimates were smaller than those made by the observers (Fig. 4). For both ERII and FIR, agreement was consistent across ROM and was similar between 2D-pose and both observers (Fig. 4).

**Table III:** Differences between observers.

Movement	Main effect of observer
	*P* value
Abduction	.001
Flexion	<.001
Extension	.981
ERI	.175
ERII	.440
FIR	.486

*ERI*, external rotation in position I; *ERII*, external rotation in position II; *FIR*, functional internal rotation.

### Aim 2: the agreement between observers

**Figure 5:**
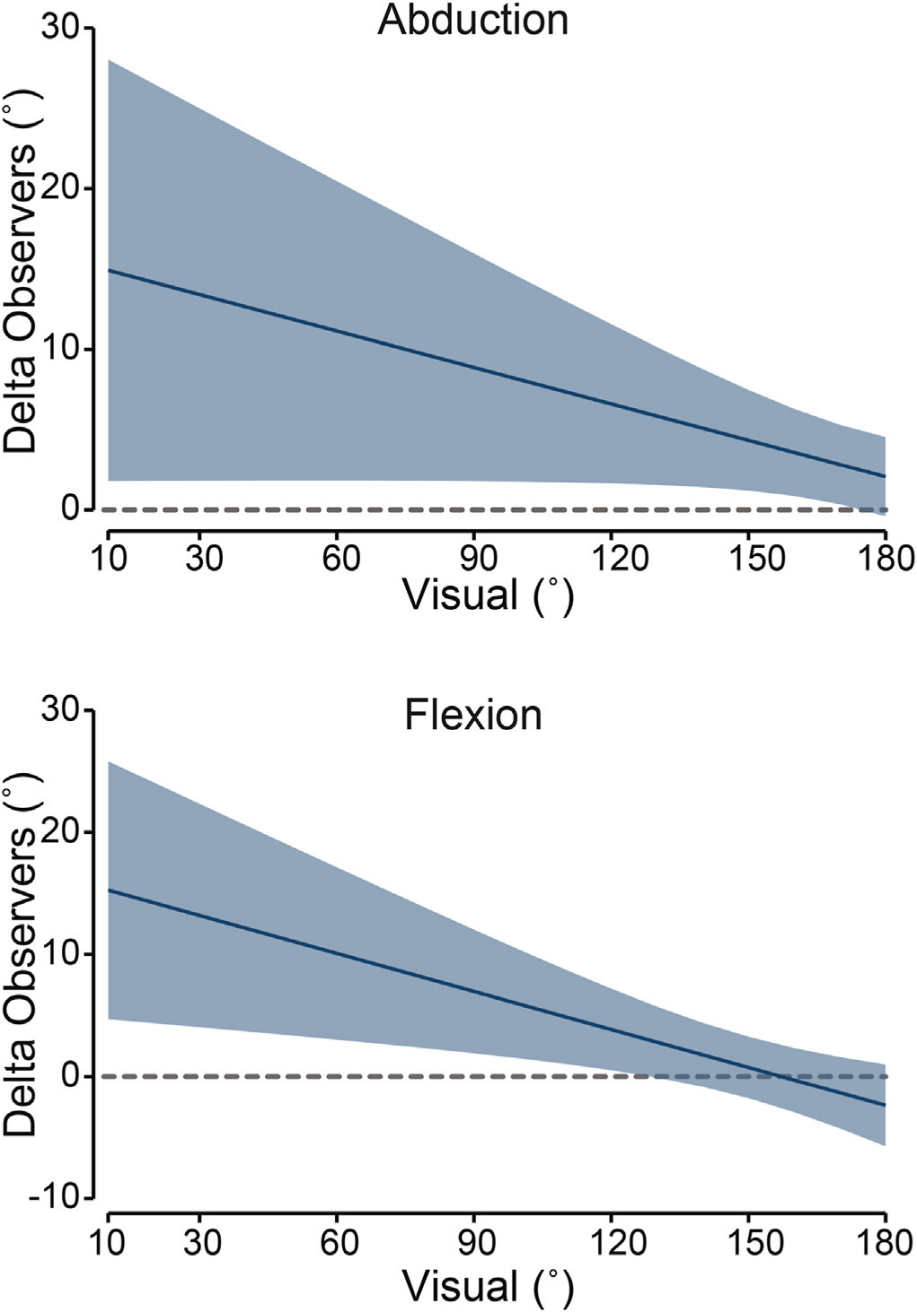
Agreement between the two observers for abduction (*top panel*) and flexion (*bottom panel*). Graphs reflect the mean difference between observers with 95% CIs (*shaded area*) derived from the mixed model (see statistical analysis for more detail). Observers agree when CI includes zero, indicated by the horizontal dashed line. *CIs*, confidence intervals.

Observers had significant disagreement for abduction and flexion ROM (visual × observer interaction: *P* < .003, Table I, Fig. 5). For abduction, the amount of disagreement ranged from 15° (95% CI: 2°, 28°) at 10° abduction, gradually decreasing to 3° (95% CI: 0.5°, 5°) at 160° flexion. Observers were in agreement for 180° abduction ROM (*P* = .22). For flexion, the amount of disagreement ranged from 15° (95% CI: 5°, 26°) at 10° flexion, gradually decreasing to 4° (95% CI: 1°, 7°) at 120° flexion, above which observers agreed (*P* > .07). For all other shoulder movements, observers agreed; neither main effect of observer (*P* > .17, Table III), nor visual estimates × observer interaction (*P* > .07, Table I) was observed.

## Discussion

The ROM estimates using 2D-pose and visual assessment were consistent for abduction, flexion, extension, and ER movements, but not for FIR. While most shoulder movements showed consistency between the two methods, they did not always agree. For abduction and flexion, this disagreement depended on the observer and the shoulder movement range performed by the participant. For extension and ERI, the disagreement between 2D-pose and observers depended only on the shoulder movement range performed by the participant. However, 2D-pose and observers agreed on ERII and FIR. In general, the SDD_95_ 95% CI was between 5° and 13° for most shoulder movements, potentially highlighting the detection resolution of visual estimates, as these estimates were mainly provided with 10° resolution (with some exceptions). Although 2D-pose and visual assessment agreed on FIR, the SDD_95_ was about 0.5 zones, underscoring the low resolution of FIR measurement by 2D-pose. These findings have potential implications for methods used to quantify efficacy of shoulder interventions. It is critical to acknowledge the potential benefits and limitations of either method in relation to the specific use case.

Pose-based methods are developed to potentially provide a more objective assessment of active shoulder ROM, aiming to standardize ROM methods.^11^ Standardized methods can better detect changes over time by eliminating inter- and intra-observer variation. They also allow for more reliable comparison between research studies that use active shoulder ROM to assess the efficacy of interventions.^16^ However, it is important to note that when there is disagreement (ie, bias) between automated methods or between observers, these methods and observers cannot be used interchangeably.^17^ Doing so would introduce variation unrelated to actual changes in a person's active shoulder ROM. Studies have confirmed that variation exists both between and within observers.^5^^,^^9^^,^^10^ Therefore, it is critical to select a method, use it consistently with patients and research participants, and acknowledge its limitations. A choice between methods can only be made when they are consistent, indicating that they measure the same construct. In our study, the variance in one method was largely accounted for by the variance in the other method for shoulder abduction, flexion, and ERII.

It is crucial to identify factors that may cause inconsistencies between methods measuring shoulder ROM, as this informs the choice of the most appropriate method. Our findings revealed inconsistencies in extension, ERI, and FIR between the 2D-pose and visual assessment methods.

Several factors could potentially explain this inconsistency for extension ROM. First, 2D-pose detection may lack sensitivity to thoracic compensation, as 2D-pose cannot accurately detect subtle thoracic movement (Fig. 1).^11^ In 2D-pose analysis, the thorax is broadly defined as the line segment from the midpoint of the hips to the midpoint of the shoulders. This broad definition reduces the method's ability to detect relative movement between these points, allowing thoracic flexion to be misinterpreted as increased shoulder extension. This limitation may explain the large variation in 2D-pose estimates for a given visual ROM estimate, as shown in the scatter plot between 2D-pose and visual assessment of extension (Fig. 4). Second, when the arm moves in front of the hip during small extension ROM, the location accuracy of the 2D-pose–detected hip landmark may be compromised, affecting the calculated relative shoulder angle. Last, shoulder extension is not commonly assessed visually in clinical practice, which may lead to increased visual observer due to unfamiliarity. However, both observers agreed on the estimated extension ROM, suggesting that observer error was not a significant factor.

Several factors may explain the inconsistency of ERI ROM estimates between 2D-pose and visual methods. Both approaches assessed ERI while viewing the participant face-on, relying on the projected forearm length to estimate shoulder ERI angle—a process that can introduce inaccuracies. In 2D-pose analysis, the forearm length is estimated from the contralateral side. The change in distance between the frontal-plane projection of the wrist and elbow is not consistent for changes in ROM, and decreases at larger ERI angles. The range-of-motion model within the mymobility App accounts for this limitation by capping ERI angle estimates above 45°. Observers also assessed ERI from a frontal view, and accuracy may improve when observers change their perspective, as demonstrated by Wilson et al.^24^ However, even when observers moved to different positions in that study, substantial variation remained between visual estimates and 3D motion capture measurements of ERI.^24^ Pose estimation methods using depth cameras (eg, Microsoft Kinect or Azure) may offer greater accuracy than single-camera 2D-pose methods.^24^ Depth cameras can detect body segments in 3D, though they require a personal computer to process the data. Taken together, both 2D-pose and visual methods are prone to inaccuracies when estimating ERI ROM, depth sensing offering potential improvement.

Several factors may explain the inconsistency in FIR ROM estimates between 2D-pose and visual methods. The 2D-pose method divides the back into four zones, which lowers the resolution of FIR estimates. Since each zone spans a range in vertebral levels, this approach reduces consistency with visual estimates. This limitation is reflected in the large variation observed in the FIR scatter plot between 2D-pose and visual assessment (Fig. 4). Accurate visual estimation of vertebral levels is also challenging and requires training. Observers assessed vertebral levels from the same position used for other shoulder movements, and participants wore clothing, which may have further reduced accuracy. Visual estimation might be more precise if the distance between the participant's thumb and sacrum were measured using a tape measure.^2^ Similarly, the resolution and potential accuracy of the 2D-pose estimates could improve if the position of the wrist were expressed as a percentage of the distance between the hip and shoulder landmarks. However, this approach requires further investigation. For research purposes, if FIR were to be used as an outcome measure, neither the 2D-pose nor visual method appears to provide sufficient accuracy. This underscores the need for further refinement and development of both methods to ensure reliable and accurate assessments of FIR.

Another study showed that visual estimates tend to be more variable than the 2D-pose method. For example, the 2D-pose method showed a more consistent relationship with 3D motion capture than when compared to visual estimates.^24^ This greater variability in visual observation was evident for movements such as abduction, flexion, ERI, and internal rotation at 90° shoulder abduction.^24^ These findings suggest that automated methods are likely more accurate than visual estimates. A large variation of 2D-pose–based ROM was observed for a given visual estimate, possibly reflecting the lower resolution of visual methods compared to automated approaches.^24^ Similarly, we observed variability in visual estimates for a given 2D-pose–based shoulder ROM, as shown by the vertical distributed data points in the scatter plots between 2D-pose and visual estimates (Fig. 4).

Visual variation may impact functional shoulder scores, such as the Constant-Murley^6^ and University of California–Los Angeles score.^13^ Both assessments rely on accurate ROM measurements, and inconsistencies between visual and automated methods could undermine the reliability of these functional evaluations. Automated methods may provide a more precise alternative for evaluating shoulder movements, leading to more accurate and consistent functional shoulder scores.

Gauci et al^8^ compared visual assessments with an automated pose-based shoulder ROM method that utilized a Microsoft Kinect depth camera. Similar to our findings, they reported inconsistencies in ERI measurements. Also consistent with our findings, they observed consistency between visual assessment and their pose-based method for flexion and abduction, with some disagreement (bias) between methods.^8^ However, their method demonstrated greater consistency in measuring extension compared to ours.^8^ The 3D-pose method employed by Gauci et al^8^ may better account for compensatory movements in other segments. In addition, Gauci et al^8^ averaged the measurements from two visual observers, potentially reducing variation in their visual assessments. In contrast, we preserved the individual data from both visual assessors and considered the variation between them.

Fan et al^7^ compared shoulder abduction and flexion ROM using the OpenPose tracking algorithm^3^ against screen-based goniometric measurements. They reported lower correlations between the two methods for abduction and flexion compared to our findings, with the OpenPose method detecting larger angles than screen-based goniometry.^7^ The OpenPose method utilizes landmarks similar to those detected by the mymobility App, indicating potential variability in screen-based goniometry. Overall, the consistency between our 2D-pose–based and visual assessment appears greater than the consistency observed between OpenPose and screen-based goniometry.^7^

Our study has several limitations that require consideration. First, we included participants with typical, pain-free shoulder function, which limits the external validity of our findings. While this cohort provides an ideal population for methodological testing, individuals with shoulder pathologies may exhibit different movement strategies, including compensatory movements in adjacent segments such as the thorax. To ensure broader applicability, future studies should evaluate these methods in clinical populations and consider refining thorax reference-frame definitions to better detect and account for compensatory movements. Second, visual estimates were obtained from a fixed observer position (Fig. 3). In clinical practice, surgeons often adjust their perspective to optimize visual estimates. We fixed the observers' positions to prevent interference with 2D-pose estimates, allowing for concurrent shoulder assessment. Third, our study included a limited number of participants. However, we addressed this by collecting multiple observations by using three phones and assessing six ROM estimates for each movement per participant. To avoid inflating correlations through data duplication, we accounted for the nested nature of the data in our statistical analyses. Fourth, we did not assess the test-retest reliability of the ROM assessment methods as this was not a predefined aim of the study. While reliability testing is recommended by COnsensus-based Standards for the selection of health Measurement INstruments, it requires that the underlying construct being measured remains stable over time. This may be particularly challenging for active shoulder ROM, where even small variations in volitional effort, posture, or movement strategy can lead to meaningful changes in measured range. Interpreting test-retest reliability must consider the inherent variability of the movement performance itself, not just the measurement method. Future studies should explore the temporal stability of both 2D-pose and visual methods under controlled conditions. Finally, we ensured that shoulder movements were performed within the same plane of the 2D-camera to minimize errors caused by out-of-plane movements. Errors in 2D-pose estimates increase substantially when arm movements deviate from the camera plane.^11^ It is important to note that 2D-pose method is not suitable for assessing multiplanar or complex shoulder movements that extend outside the camera's 2D plane of view. Therefore, careful camera setup—ensuring the phone is upright and aligned with gravity, maintaining appropriate distance to capture the full upper limb, and providing clear instructions to guide planar movement—is essential for maximizing accuracy and minimizing compensatory segmental motion when using 2D-pose for shoulder ROM assessment.

## Conclusion

The 2D-pose–based estimates of abduction, flexion, and ERII compared well with visual estimates. However, discrepancies emerged in agreement between the visual observers for abduction and flexion, underscoring the value of automated methods in reducing inter-rater variability. In contrast to abduction, flexion, and ERII, estimates for ERI, extension, and FIR were less consistent between visual and 2D-pose assessment methods. These findings highlight the need to carefully consider the limitations of both 2D-pose and visual methods when quantifying these movements.

## Disclaimers:

Funding: This research was funded by the Australian Research Council Industrial Transformation and Training Centre for Joint Biomechanics (IC190100020) of which Zimmer Biomet is an industry partner.

Conflicts of interest: Kenneth Cutbush reports a relationship with Stryker that includes: consulting or advisory and funding grants; a relationship with Arthrex that includes: consulting or advisory and funding grants; a relationship with Johnson & Johnson that includes: consulting or advisory; a relationship with Device Technologies that includes: funding grants; reports share ownsership in Tetrous; and gratefully acknowledges funding from the Australian Research Council through the Industrial Transformation Training Centre for Joint Biomechanics (IC190100020) and its associated industry partners; cash contributing partners include Stryker, Zimmer Biomet, Logemas, and Australian Biotechnologies. Ashish Gupta is CEO of Akunah Medical Technology Pty Ltd.; he is a consultant for Zimmer Biomet, Device Technologies; he is the codirector of the Australia Shoulder Research Institute which received funding from Stryker, Zimmer Biomet, Device Technologies and Arthrex; he is the founding director of Queensland Unit for Advanced Shoulder Research at the Queensland University of Technology which receives funding form the Australian Research Council (Grant ID IC190100020), QUT, Stryker, Zimmer Biomet, Australian Biotechnologies, Materialise and Akunah; and he has stock options in Akunah Medical Technology Pty Ltd. and Tetrous Inc. The other authors, their immediate families, and any research foundation with which they are affiliated have not received any financial payments or other benefits from any commercial entity related to the subject of this article.
